# Comparison of automated ultrasound pupillometry assessment vs. infrared pupillary in critically ill patients

**DOI:** 10.3389/fmed.2025.1722645

**Published:** 2026-01-12

**Authors:** Weiting Chen, Xiaoshuang Jiang, Xixi Guo, Jiuzhou Lin, Nanlin Dou, Min Tang

**Affiliations:** 1 Department of Emergency Medicine, The First People's Hospital of Linhai, Taizhou, Zhejiang, China; 2 Department of Traditional Chinese Medicine, The First People's Hospital of Linhai, Taizhou, Zhejiang, China; 3 Department of Intensive Care Medicine, Lishui Traditional Chinese Medicine Hospital Affiliated to Zhejiang Chinese Medical University, Lishui, Zhejiang, China

**Keywords:** infrared pupillometry, neurocritical care, pupillary light reflex, ultrasound, ultrasound pupillometry

## Abstract

**Background:**

The pupillary light reflex (PLR) is a critical indicator of brainstem function in neurocritical care, but traditional assessments are often subjective, inconsistent, and prone to inter-examiner variability. Automated infrared pupillometry (IPA) provides objective metrics and is increasingly used in clinical practice, yet its accuracy may be compromised by eyelid swelling or ocular trauma. Automated ultrasound pupillometry (Auto-UPA) offers a non-invasive, transpalpebral alternative that can overcome these limitations. This study aimed to evaluate the agreement and reliability of Auto-UPA compared with IPA in ICU patients.

**Methods:**

In this prospective observational study, consecutive adult ICU patients (February 1 to September 25, 2025) underwent paired Auto-UPA and IPA assessments under standardized conditions. PLR metrics included initial (INIT) and end (END) pupil diameters, latency (LAT), constriction ratio (DELTA), average constriction velocity (ACV), and average dilation velocity (ADV). Agreement was assessed using linear regression, Bland-Altman plots, and limits of agreement (LoA). Reliability was evaluated using intraclass correlation coefficients (ICC) for intra- and inter-observer variability.

**Results:**

Twenty patients (40 eyes) were enrolled. High agreement was observed for pupil diameters and most dynamic indices (INIT: *R*^2^ = 0.989, bias = 0.01 mm, LoA = −0.11 to 0.12 mm; END: *R*^2^ = 0.992, bias = 0.02 mm, LoA = −0.07 to 0.11 mm), DELTA (*R*^2^ = 0.980, bias = −0.5%, LoA = −2.1 to 1.1%), ACV (*R*^2^ = 0.960, bias = −0.19 mm/s, LoA = −0.31 to −0.06 mm/s), ADV (*R*^2^ = 0.899, bias = −0.04 mm/s, LoA = −0.15 to 0.06 mm/s). LAT demonstrated only moderate concordance (*R*^2^ = 0.550, bias = 0.12 s, LoA = 0.08–0.16 s). Inter-observer ICCs ranged from 0.940 (LAT) to 0.999 (END), while intra-observer ICCs ranged from 0.890 (LAT) to 0.985 (END), indicating good-to-excellent reliability across all parameters.

**Conclusions:**

Auto-UPA shows strong agreement with IPA in most PLR metrics and provides robust reliability, supporting its role as a feasible and practical alternative in settings where IPA is unavailable or impractical.

## Introduction

Neurological examination is a cornerstone of bedside assessment in acute brain injury, offering immediate and actionable insights. The pupillary light reflex (PLR) provides a rapid, non-invasive indicator of brainstem integrity and intracranial pathology. Because of its simplicity and clinical value, PLR monitoring has become an integral part of neurocritical care. Consistent neuromonitoring is crucial for detecting cerebral changes and guiding interventions to prevent secondary injury (1). Deterioration in PLR is linked to poorer outcomes in acquired brain injury (2–4). However, manual visual assessments are highly subjective and prone to variability, influenced by ambient lighting, examiner experience, visual acuity, and stimulus inconsistencies, which underscoring the need for more objective and standardized methods (5).

Automated pupillometry delivers rapid, reliable, and quantitative PLR evaluation, thereby aiding both diagnosis and decision-making (6, 7). Infrared pupillometry (IPA) has already transitioned into routine clinical use, with indices such as the Neurological Pupil index (NPi) predicting neuro-worsening and the need for care escalation in traumatic brain injury (TBI) (8). In blunt TBI, an admission NPi < 3 predicts intensive care needs, and combining it with transcranial Doppler further enhances diagnostic accuracy (9). Despite these advantages, IPA remains relatively inaccessible outside specialized centers and is often impractical in cases of severe eyelid edema or orbital trauma, both of which are common in critically ill patients.

B-mode ultrasound, widely available and non-invasive, enables transpalpebral ultrasonographic pupillary assessment (UPA) when optical access is restricted, while still adhering to ophthalmic safety standards. In ICU settings, UPA correlates strongly with IPA for pupil diameters (*r* ≈ 0.93–0.97; error ≈ 2%−3%), suggesting potential interchangeability (10). Ongoing ultrasound advancements also support a range of ocular applications, including trauma evaluation and foreign body detection (11, 12). Nevertheless, manual UPA has key limitations: it requires a second operator to provide light stimuli and immediate post-freeze measurements, which reduces efficiency, increases operator dependence, and restricts its widespread use (13).

Automated UPA (Auto-UPA) integrates illumination, automated capture, pupil recognition through closed eyelids, edge tracking, and generates outputs such as diameter, latency, average constriction velocity (ACV), and average dilation velocity (ADV), thereby making it scalable where IPA is unavailable. Recent work has shown near-perfect agreement between automated and manual UPA (*r* ≈ 0.996–0.999) while offering faster acquisition (14). However, its comparative performance relative to IPA remains undercharacterized. Therefore, the present prospective study was designed to assess the agreement and reliability of Auto-UPA vs. IPA across key PLR metrics in ICU patients.

## Methods

### Study design and ethics

This prospective observational study adhered to the Declaration of Helsinki and was approved by the Institutional Review Board of The First People's Hospital of Linhai (approval No. 2024009). Consecutive adults (≥18 years) admitted to the ICU between February 1 and September 25, 2025, were enrolled. Patients were excluded if they had ophthalmologic or periorbital conditions that could confound pupillary assessment (e.g., atypical pupil anatomy, prior ocular surgery, or severe pre-existing neurological deficits, such as prior severe brain injury resulting in a chronic disorder of consciousness with a Glasgow Coma Scale ≤ 8, that precluded meaningful evaluation).

### Informed consent and data collection

Informed consent was obtained from legally authorized representatives for all patients who lacked decision-making capacity due to acute critical illness, altered sensorium including those with Glasgow Coma Scale (GCS) scores indicating moderate brain injury, sedation, or mechanical ventilation. For patients who were fully alert, oriented, and demonstrated decision-making capacity, written informed consent was obtained directly from the patient. The GCS was used to assess neurologic severity at enrollment. Demographics, comorbidities, vital signs, and hemodynamic parameters were extracted from electronic medical records.

To ensure standardization, non-invasive Auto-UPA and IPA assessments were performed sequentially during the same visit under controlled dim lighting (lights off, curtains closed). Auto-UPA was always conducted first, followed within 3 min by IPA, with no intervening procedures to minimize bias. Both eyes were examined when feasible, and three acquisitions per eye per modality were averaged to enhance repeatability. Examinations were conducted throughout ICU stays by three trained investigators (C.Y., S.P., and M.L.), and in a subset of 20 patients, repeated sessions were performed to evaluate measurement stability over time. For the reliability analysis, two of the three investigators independently acquired three consecutive Auto-UPA measurements per eye within the same session from these 20 patients, allowing quantification of both inter- and intra-observer agreement.

### Devices

**Figure 1:**
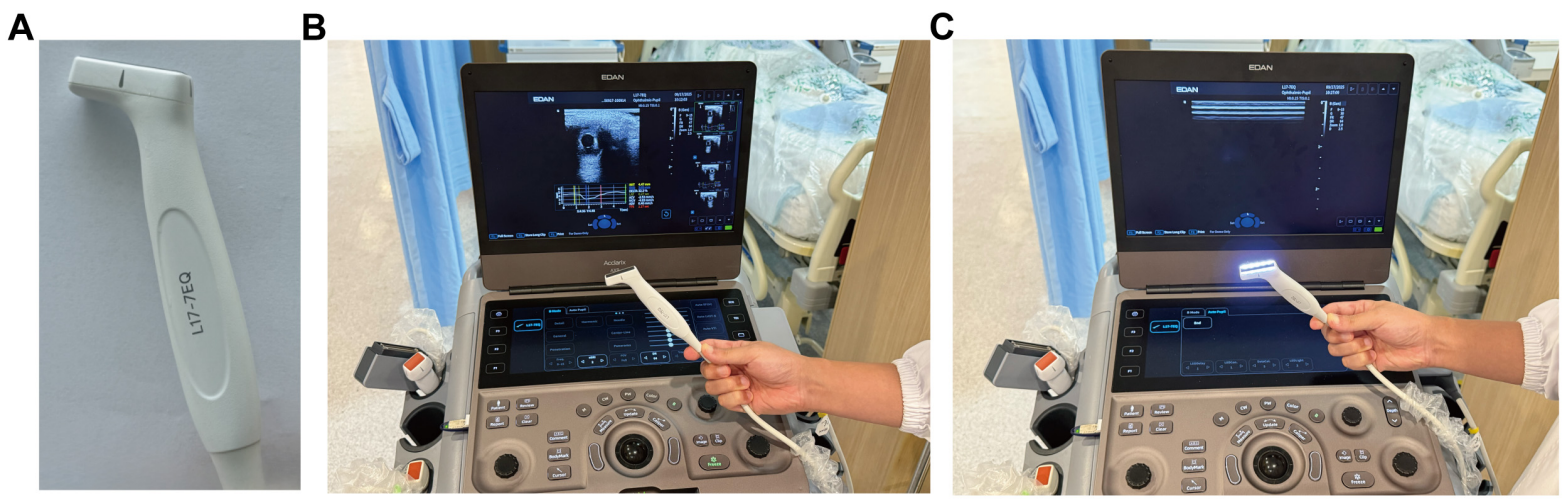
Automated ultrasound pupillometry system. **(A)** Handheld linear ocular probe (L17–7EQ) used for transpalpebral imaging. **(B)** EDAN AX8 ultrasound system in the “Ophthalmic-Pupil” preset, displaying the B-mode ocular image. **(C)** Auto-UPA pupillometry mode with the integrated light source activated, ready for automated pupillary light reflex acquisition.

**Figure 2:**
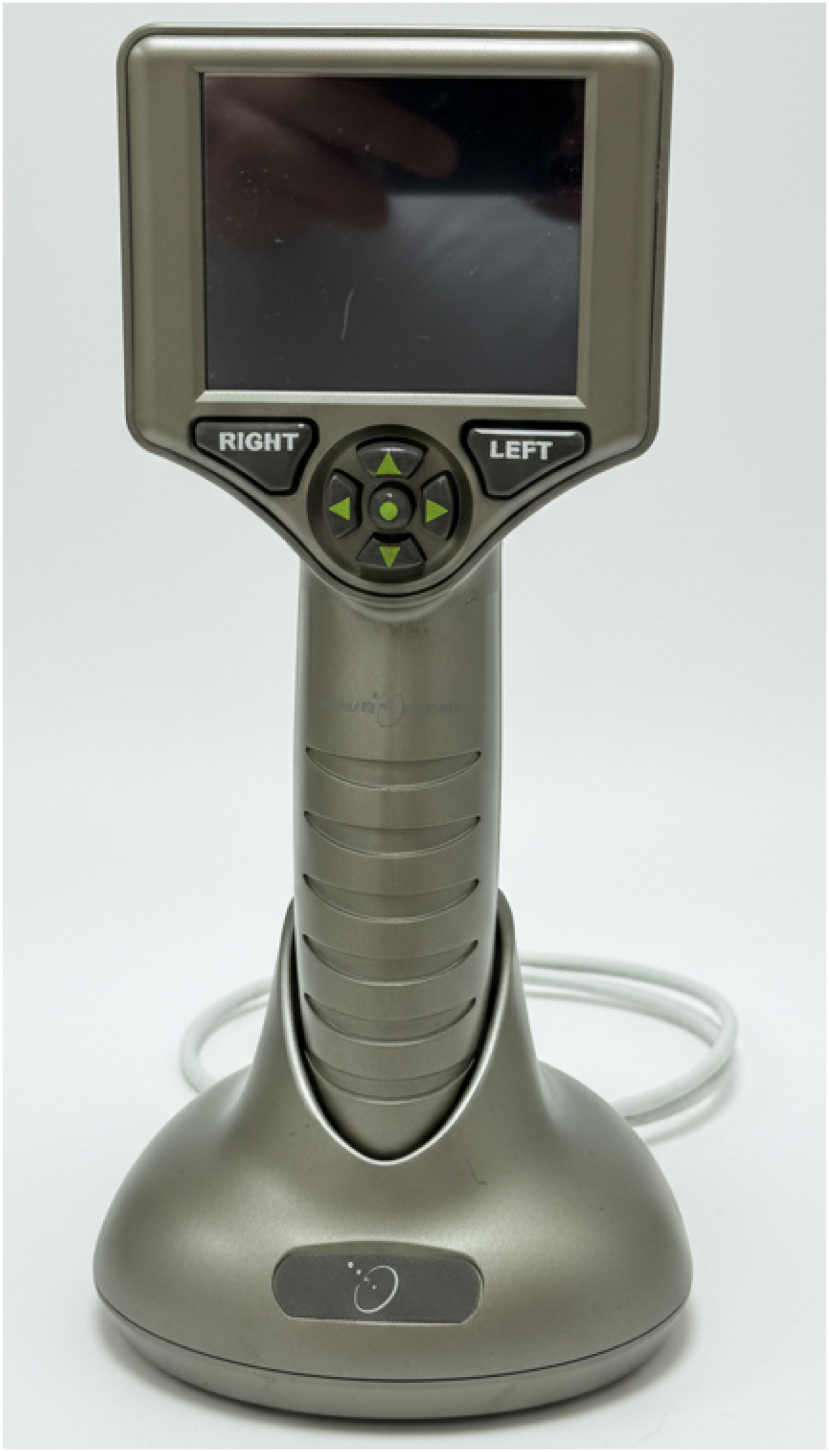
Monocular infrared pupillometer. Clinically approved infrared pupillometer (NPi-300) used for standard quantitative pupillary assessment in this study.

Auto-UPA was performed using the EDAN AX8 ultrasound system (Edan Instruments, Shenzhen, China) equipped with a handheld ocular probe on the “Ophthalmic-Pupil” preset, operated strictly within safety limits (Figure 1). IPA (NPi-300) employed a clinically approved monocular infrared pupillometer (Figure 2). Both devices were used in accordance with routine clinical care protocols and required no hardware modifications.

### Auto-UPA

**Figure 3:**
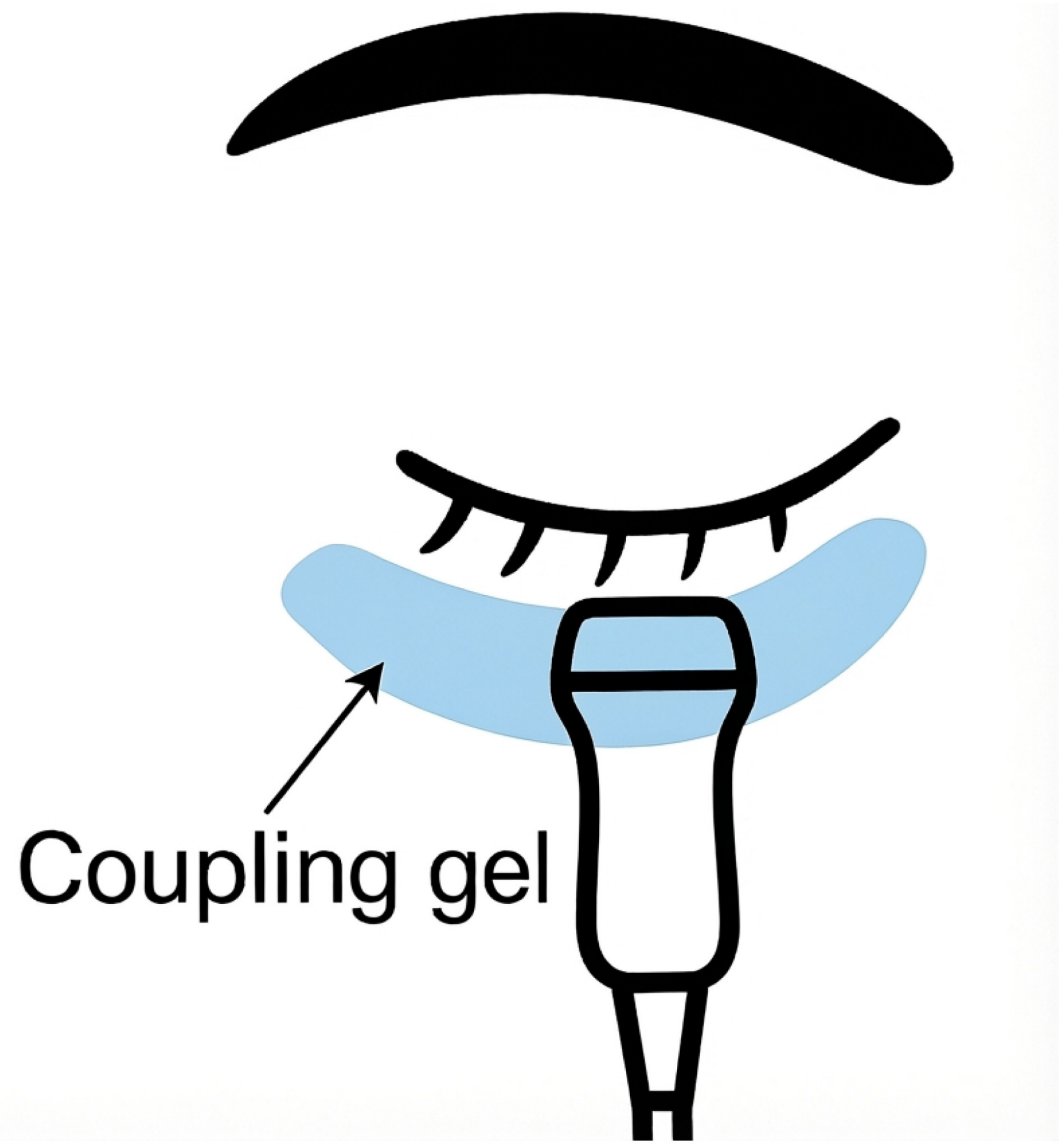
Schematic of transpalpebral probe placement for Auto-UPA. Transverse placement of the linear ocular transducer over the closed lower eyelid with a thin, continuous layer of coupling gel applied along the entire lower lid. Only light, uniform pressure is applied to obtain a tangential view of the pupil while maintaining ocular safety.

Bilateral pupillary diameter and light reflex were evaluated using a standardized automated ultrasonographic protocol. With the console set to the “Ophthalmic-Pupil” preset, patients were positioned in either the supine or semi-recumbent posture. A linear 7.5–15 MHz ocular transducer (EDAN AX8, Edan Instruments, Shenzhen, China) was placed trans-palpebrally over the closed eyelid at the inferior margin. To ensure standardization and reproducibility, the probe was placed in a transverse orientation with the probe marker strictly oriented toward the patient's right side for all acquisitions, achieving a tangential pupil view. Examinations adhered to ophthalmic ultrasound safety guidelines and the ALARA (As Low As Reasonably Achievable) principle, with the preset acoustic power applied. With the eyelid gently closed, a thin, continuous layer of coupling gel was evenly applied along the entire lower eyelid, and only light, uniform contact pressure was maintained (Figure 3).

Following probe placement in a transverse orientation over the lower eyelid, the transducer was gently adjusted until the pupil margin and anterior segment were clearly visualized. For each eye, with the eyelid gently closed, a standardized ipsilateral light stimulus was delivered trans-lid using the probe's integrated light source to elicit the direct PLR. The system's pupillometry mode then captured the dynamic diameter trace (time–diameter curve).

**Figure 4:**
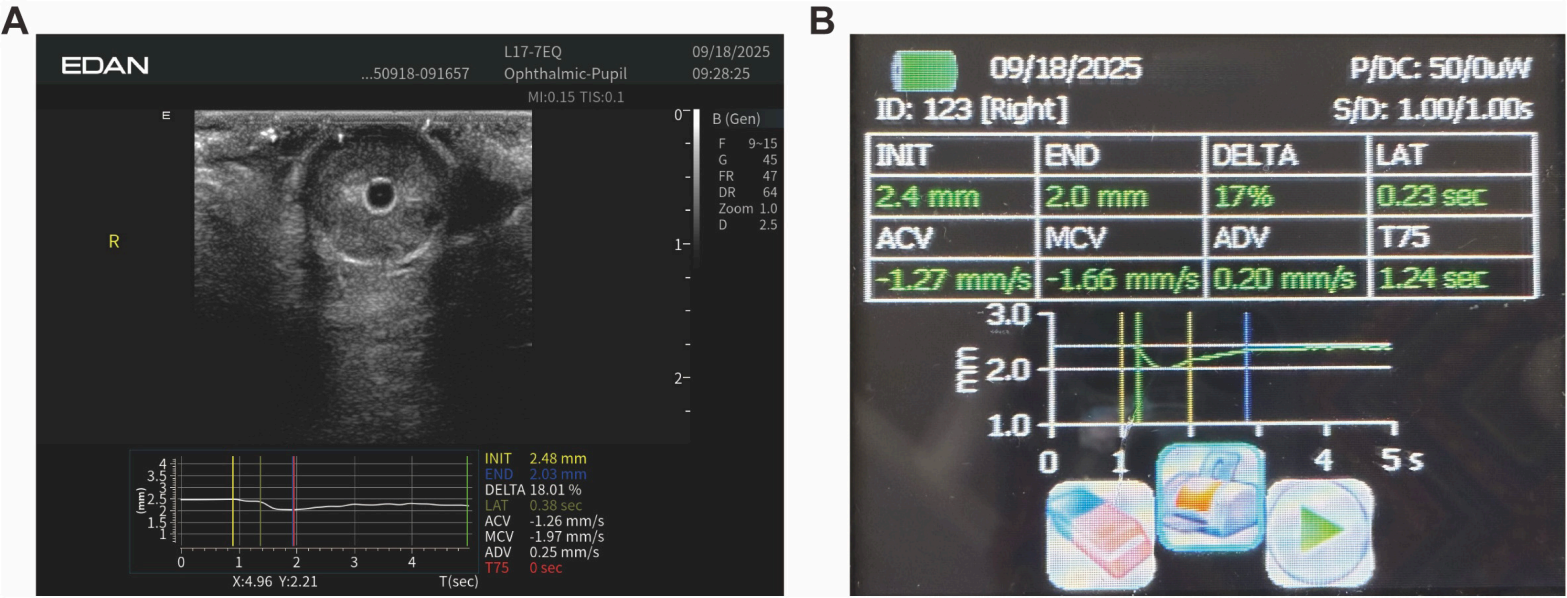
Representative Auto-UPA acquisition and on-screen output. **(A)** B-mode ocular image with overlaid time–diameter trace and automatically derived pupillary light reflex metrics, including initial diameter (INIT), end diameter (END), constriction ratio (DELTA), latency (LAT), average constriction velocity (ACV), and average dilation velocity (ADV). **(B)** Corresponding Auto-UPA summary screen for the same eye, displaying the numerical values of PLR metrics in a tabular format.

The Auto-UPA algorithm processed each B-mode cine loop through a standardized pipeline. First, a short pupillary cine sequence was acquired under fixed depth and gain settings. Second, the pupil region of interest was automatically localized using intensity-based thresholding and edge detection, followed by morphological filtering to segment a smooth pupil boundary. Third, pupil diameter was computed frame by frame as the maximal horizontal chord through the pupil centroid, generating a continuous diameter–time curve aligned to the onset of the light stimulus. Finally, key PLR metrics were automatically derived and exported for analysis, including initial diameter (INIT), end constriction diameter (END), constriction ratio (DELTA = [INIT – END]/INIT), latency (LAT; time from light onset to the onset of constriction), average constriction velocity (ACV), and average dilation velocity (ADV). On-screen quality-control prompts verified adequate signal acquisition (Figure 4A).

To enhance repeatability and minimize within-eye variability, three consecutive acquisitions were performed per eye within a single session (total acquisition time ≈30 s per eye), and the results were averaged arithmetically. The measurement sequence was fixed (Auto-UPA always preceding IPA) to minimize systematic bias.

Although Auto-UPA was considered minimal risk, operators monitored for any transient eye discomfort, irritation, redness, or pain related to probe pressure or gel application, and all adverse events were documented. All acquisitions were performed under the same standardized lighting conditions described above by the three trained investigators.

### IPA

IPA was conducted immediately after Auto-UPA using a standard monocular infrared pupillometer. The device was carefully positioned over the open eye, and a calibrated light stimulus was applied trans-corneally to elicit the PLR. All key parameters were automatically calculated by the device using its proprietary algorithms to align with the Auto-UPA output for direct comparison (Figure 4B).

### Statistical analysis

Continuous baseline variables were summarized as mean ± SD for normally distributed data or median (IQR) for non-normally distributed data, and were compared between groups using Student's *t*-test or the Mann–Whitney *U*-test, as appropriate. Paired, eye-level comparisons between Auto-UPA and IPA were performed using the per-eye average of three consecutive acquisitions. Agreement was assessed with Bland–Altman plots (bias and 95% limits of agreement), while linear association was quantified using ordinary least-squares regression (slope, intercept, *R*^2^) and Pearson's *r*, with 95% CIs calculated via Fisher's z transformation. For the Auto-UPA reliability subset, inter-observer agreement was quantified using intraclass correlation coefficients [ICC (2,1); two-way random-effects, absolute agreement] based on the mean of three repeated measurements per eye obtained independently by two investigators, whereas intra-observer repeatability was assessed using ICC (3,1; two-way mixed-effects, consistency) derived from three within-operator repetitions per eye. Normality assumptions were tested with the Shapiro–Wilk test and visually inspected using Q–Q plots. Categorical variables were compared using the Chi-square test or Fisher's exact test, as appropriate. All tests were two-sided with α = 0.05. Statistical analyses were conducted using GraphPad Prism 9 (v9.5.1) and Python 3.10 (SciPy/NumPy/statsmodels).

## Results

### Patient demographics and clinical characteristics

We enrolled 20 critically ill patients with neurological pathology (65% male), aged 18–84 years (mean 53.5 years). At enrollment, neurologic severity as measured by the GCS averaged 8 (range 3–15), reflecting substantial heterogeneity in the level of consciousness. A standardized Auto-UPA protocol was applied to both eyes, yielding 40 eye-level observations (20 right, 20 left). Each eye was measured three times in the same session, and the arithmetic mean was analyzed to enhance repeatability and reduce within-eye noise, thereby stabilizing physiologic estimates and aligning with best practices for reliability-sensitive metrics.

### INIT and END

Shapiro–Wilk tests indicated that both INIT and END pupil diameters were approximately normally distributed for Auto-UPA and IPA (all *p* > 0.05; Table 1). Accordingly, INIT and END values are reported as mean ± SD. Table 1 summarizes the distribution of INIT and END for each modality, including sample size, mean ± SD, and Shapiro–Wilk *p* values. Initial pupil diameters were virtually identical between modalities (Auto-UPA 4.05 ± 0.58 mm vs. infrared 4.05 ± 0.57 mm). End diameters were also closely matched (Auto-UPA 3.05 ± 0.47 mm vs. infrared 3.02 ± 0.45 mm), supporting overall concordance in absolute pupil size measurements between the two techniques.

**Table 1:** Distribution of initial (INIT) and end (END) pupil diameters measured by Auto-UPA and IPA.

**Parameter**	**Modality**	***n* (eyes)**	**Mean ±SD, mm**	**Shapiro–Wilk *p***
Initial pupil diameter (INIT)	Auto-UPA	40	4.05 ± 0.58	0.067
Initial pupil diameter (INIT)	Infrared pupillometry	40	4.05 ± 0.57	0.126
End pupil diameter (END)	Auto-UPA	40	3.05 ± 0.47	0.437
End pupil diameter (END)	Infrared pupillometry	40	3.02 ± 0.45	0.532

**Figure 5:**
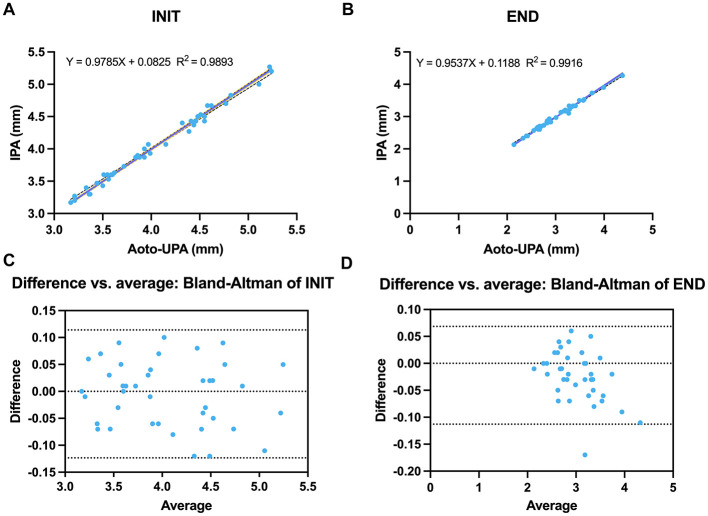
Agreement between Auto-UPA and IPA for pupil diameters. **(A)** Linear regression of initial pupil diameter (INIT) measured by Auto-UPA vs. IPA. **(B)** Linear regression of end pupil diameter (END) for Auto-UPA vs. IPA. **(C)** Bland–Altman plot showing the difference vs. average for INIT. **(D)** Bland–Altman plot showing the difference vs. average for END. The dashed lines indicate mean bias and 95% limits of agreement.

Auto-UPA and IPA demonstrated excellent linear agreement for both maximum (INIT) and minimum (END) pupil diameters. Fitted regressions for INIT (*Y* = 0.9785*X* + 0.0825; *R*^2^ = 0.9893) and END (*Y* = 0.9537*X* + 0.1188; *R*^2^ = 0.9916) yielded slopes close to unity with small intercepts, indicating near-identity across the measurement range (Figures 5A, B). Bland–Altman analyses (Auto-UPA – infrared) showed a negligible mean bias for INIT (0.005 mm) with narrow 95% limits of agreement (−0.114 to 0.123 mm), and a small positive bias for END (0.022 mm; 95% limits of agreement −0.068 to 0.113 mm). Regression of the differences on the within-pair means demonstrated no evidence of proportional bias for INIT (β = 0.016, *p* = 0.333). For END, the slope was statistically different from zero (β = 0.043, *p* = 0.006); however, the corresponding change in bias across the observed diameter range was < 0.1 mm, which we considered clinically negligible (Table 2). Taken together, these findings support the practical interchangeability of Auto-UPA and IPA for both INIT and END (Figures 5C, D).

**Table 2:** Agreement between Auto-UPA and IPA for pupillary light reflex metrics (Auto-UPA – infrared).

**Metric**	**Auto-UPA mean ±SD**	**Infrared mean ±SD**	**Mean bias**	**95% limits of agreement**	**Pearson *r***
INIT (mm)	4.055 ± 0.583	4.050 ± 0.574	0.005	−0.114 to 0.123	0.99
END (mm)	3.047 ± 0.467	3.024 ± 0.447	0.022	−0.068 to 0.113	0.996
DELTA (fraction)	0.247 ± 0.051	0.252 ± 0.052	−0.005	−0.021 to 0.011	0.988
LAT (s)	0.399 ± 0.030	0.278 ± 0.027	0.121	0.080–0.161	0.742
ACV (mm/s)	1.274 ± 0.271	1.461 ± 0.297	−0.186	−0.309 to −0.063	0.980
ADV (mm/s)	0.732 ± 0.166	0.776 ± 0.173	−0.044	−0.152 to 0.064	0.948

### Constriction ratio (DELTA) and latency (LAT)

**Figure 6:**
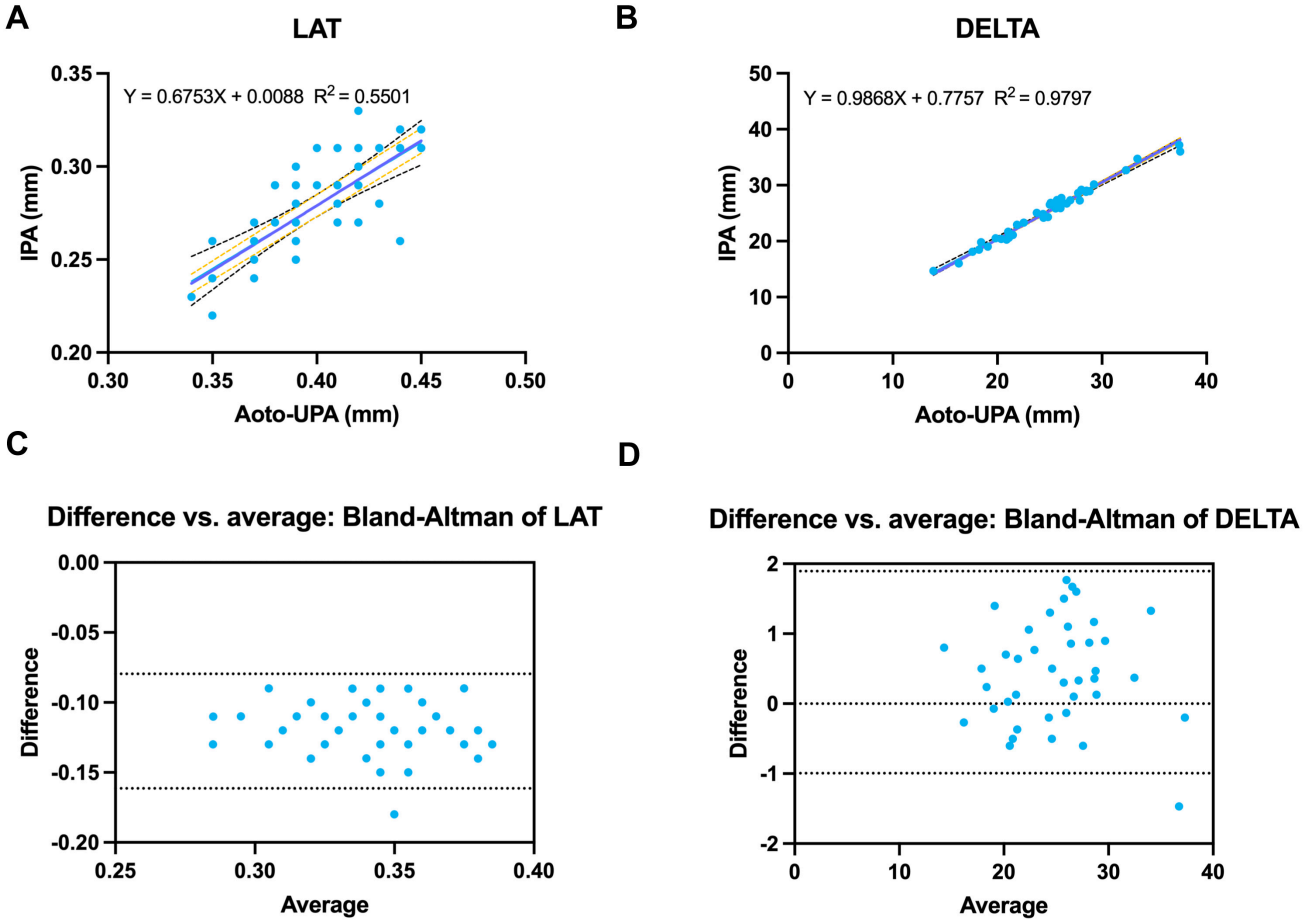
Agreement between Auto-UPA and IPA for latency and constriction ratio. **(A)** Linear regression of constriction latency (LAT) measured by Auto-UPA vs. IPA. **(B)** Linear regression of constriction ratio (DELTA) for Auto-UPA vs. IPA. **(C)** Bland–Altman plot of LAT (difference vs. average). **(D)** Bland–Altman plot of DELTA (difference vs. average). Dashed lines represent mean bias and 95% limits of agreement, illustrating greater between-method dispersion for LAT than for DELTA.

Auto-UPA and IPA exhibited strong agreement for DELTA but only moderate agreement for LAT. In method-comparison plots, DELTA displayed a near-unity slope with a very high coefficient of determination (*Y* = 0.9868*X* + 0.7757; *R*^2^ = 0.9797), reflecting excellent concordance across the full range of values. By contrast, LAT showed a shallower slope and lower explained variance (*Y* = 0.6753*X* + 0.0088; *R*^2^ = 0.5501), indicating greater between-method dispersion (Figures 6A, B). Constriction ratios were highly similar between modalities (Auto-UPA 0.247 ± 0.051 vs. infrared 0.252 ± 0.052; Table 2). Bland–Altman analysis (Auto-UPA – infrared) showed a negligible mean bias of −0.005 with narrow 95% limits of agreement (−0.021 to 0.011) and excellent linear association (*r* = 0.99), indicating only modest between-method dispersion for DELTA. In contrast, latency exhibited systematically higher values with Auto-UPA than with IPA (0.399 ± 0.030 vs. 0.278 ± 0.027 s; Table 2). The corresponding Bland–Altman plot demonstrated a mean bias of 0.121 s (Auto-UPA – infrared) with relatively wide 95% limits of agreement (0.080–0.161 s) and only moderate correlation (*r* = 0.74), consistent with greater between-method variability for this dynamic timing parameter compared with static diameter metrics (Figures 6C, D).

### Velocity metrics (ACV and ADV)

**Figure 7:**
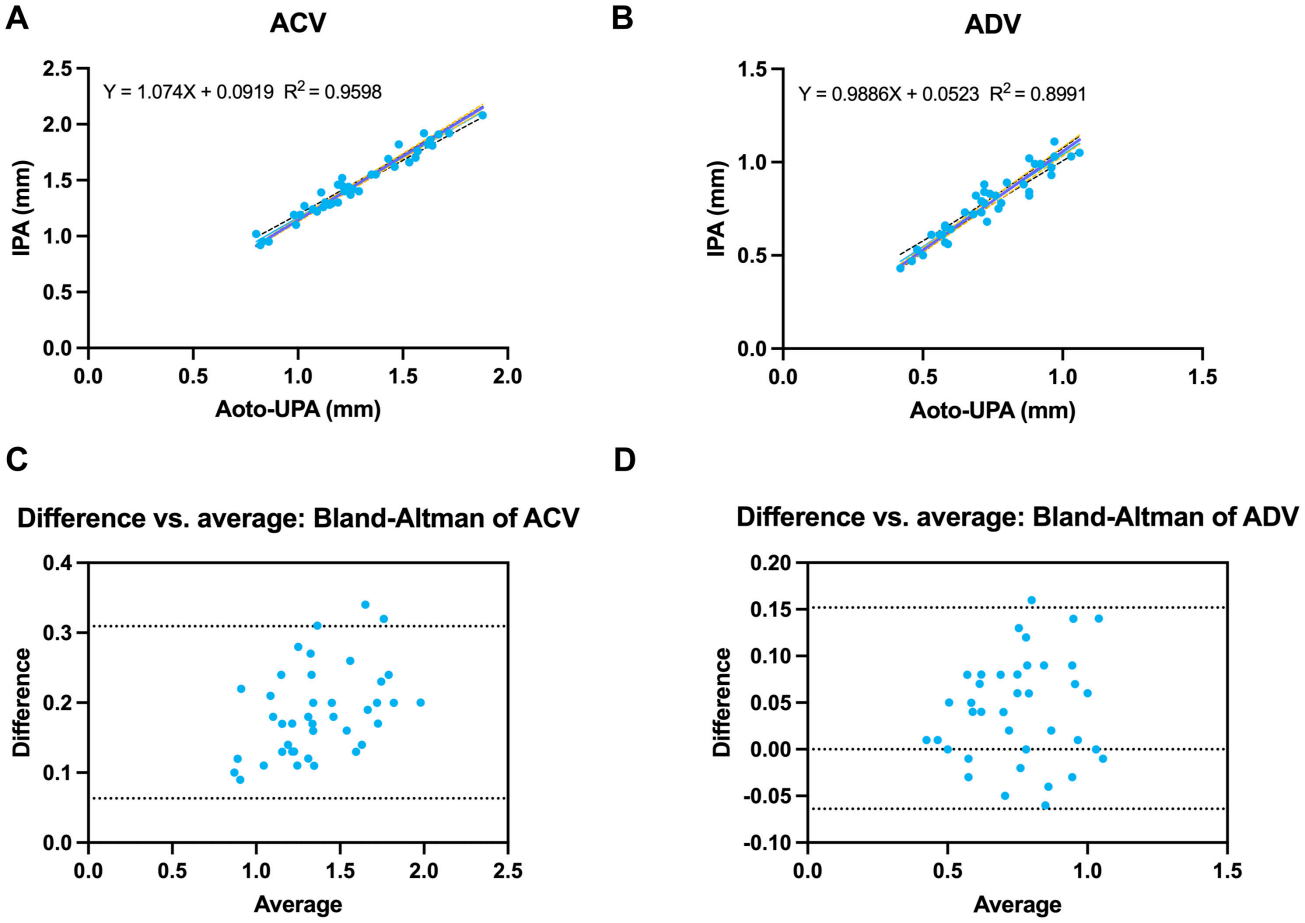
Agreement between Auto-UPA and IPA for velocity metrics. **(A)** Linear regression of average constriction velocity (ACV) measured by Auto-UPA vs. IPA. **(B)** Linear regression of average dilation velocity (ADV) for Auto-UPA vs. IPA. **(C)** Bland–Altman plot of ACV. **(D)** Bland–Altman plot of ADV. Dashed lines show mean bias and 95% limits of agreement, with ACV demonstrating slightly wider limits than ADV.

Auto-UPA and IPA showed strong agreement for both velocity metrics. In the method-comparison plots, ACV had a higher *R*^2^ (0.9598) than ADV (0.8991). ACV demonstrated a slope slightly greater than unity (1.074), suggesting a minor proportional tendency, whereas ADV was nearly identical to unity (0.9886), indicating near-perfect mapping (Figures 7A, B). ACV was slightly lower with Auto-UPA than with IPA (1.27 ± 0.27 vs. 1.46 ± 0.30 mm/s; Table 2). Bland–Altman analysis (Auto-UPA – infrared) revealed a small negative mean bias of −0.186 mm/s, with moderate 95% limits of agreement (−0.309 to −0.063 mm/s) and high linear correlation (*r* = 0.98). ADV showed a near-zero mean bias (−0.044 mm/s) with relatively narrow 95% limits of agreement (−0.152 to 0.064 mm/s) and strong correlation (*r* = 0.95), indicating closer agreement between modalities for dilation dynamics than for constriction velocity (Figures 7C, D).

### Safety

During all Auto-UPA sessions, no eye discomfort, redness, pain, or other adverse events related to probe pressure or gel application were reported or observed.

### Reliability (inter- and intra-observer ICCs)

**Figure 8:**
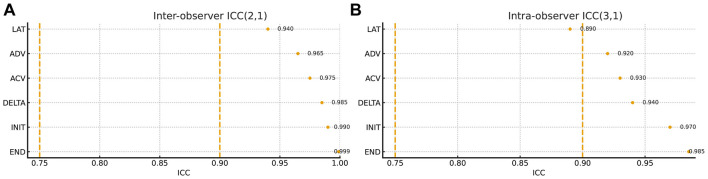
Inter- and intra-observer reliability of Auto-UPA metrics. **(A)** Inter-observer agreement for automated ultrasound pupillometry (Auto-UPA), quantified using ICC (2,1) between two independent operators based on three repeated measurements per eye. **(B)** Intra-observer repeatability for Auto-UPA, quantified using ICC (3,1) from three within-operator repetitions per eye.

Figure 8 summarizes the inter- and intra-observer reliability of Auto-UPA metrics in the predefined reliability subset. For inter-observer agreement [ICC (2,1)], Auto-UPA demonstrated excellent concordance between the two operators across all six PLR parameters, with ICC values ranging from approximately 0.94 (LAT) to 0.99 (END). INIT and END showed the highest inter-observer ICCs (≈0.990 and 0.999, respectively), while DELTA, ADV, and ACV also remained in the excellent range (all ≈ 0.965–0.975). Intra-observer repeatability [ICC (3,1)] was likewise high, with ICCs of ≈0.970 for INIT, ≈0.985 for END, ≈0.940 for DELTA, ≈0.890 for LAT, ≈0.930 for ACV, and ≈0.920 for ADV, indicating good-to-excellent short-term consistency of repeated measurements within the same operator.

Overall, diameter metrics (INIT and END) exhibited the most robust inter- and intra-observer reliability, followed by DELTA and the velocity metrics (ACV and ADV). Although LAT and particularly ADV showed comparatively lower intra-observer ICCs than the diameter-based indices, they still remained in the good-to-excellent range, underscoring the stability of most Auto-UPA–derived PLR parameters under standardized acquisition conditions.

## Discussion

In this prospective, paired ICU cohort encompassing patients with critically ill with severe neurologic pathology, we observed strong concordance between Auto-UPA and IPA across all major pupillary measures. Diameter metrics demonstrated the highest agreement, whereas dynamic indices were broadly concordant but exhibited greater variability. Collectively, these results support the use of Auto-UPA for quantitative assessment of pupil diameter and pupillary light reflex in critically ill patients when direct optical access is limited or not feasible. To our knowledge, this is the first head-to-head comparison of an automated UPA platform with IPA in the ICU, extending prior work on manual UPA and highlighting the clinical feasibility of a trans-palpebral, ultrasound-based alternative when eyelid opening is not possible.

This study further demonstrates that Auto-UPA is a rapid, non-invasive, and practical technique that yields highly reliable data relative to IPA—particularly for pupillary size and light reflex, which are central to detecting or trending brain herniation in patients with restricted ocular access. Our modality-to-modality comparison aligns with prior UPA–IPA studies, which reported strong correlation and low percentage error for diameter metrics under standardized ambient lighting and short inter-test intervals—methodological controls that were explicitly incorporated in our protocol (10). Beyond diameters, our paired analysis of dynamic metrics is consistent with broader evidence: velocity indices (ACV/ADV) are sensitive to sampling and edge-tracking parameters yet remain valid for within-device trending, whereas latency is more device-dependent because it hinges on stimulus timing and time-base synchronization—limitations repeatedly emphasized in practice-oriented reviews of automated pupillometry (7). Our finding of an approximately 0.19 mm/s bias in ACV, corresponding to about 13% of the average constriction velocity, illustrates this higher susceptibility of constriction dynamics to timing and segmentation variability but remains within a range that may be acceptable for many bedside applications. Finally, our adoption of an Auto-UPA platform is consistent with recent work showing near-identity with manual UPA while reducing acquisition time, thereby supporting its feasibility in scenarios where infrared devices are unavailable or optical access is restricted (14). In line with this, Auto-UPA demonstrated good-to-excellent inter- and intra-observer reliability across all PLR metrics, with ICC values consistently above 0.88 and approaching 0.99 for diameter-based indices. In sum, our results position automated ultrasound pupillometry as both a credible substitute when infrared access is limited and a complementary component of multimodal neuro-monitoring strategies that already include IPA alongside optic nerve sheath diameter and transcranial Doppler (15).

In neurocritical care, Auto-IPA has repeatedly demonstrated clinical utility for triage and monitoring. Case-series and cohort studies have consistently linked quantitative pupillary metrics with transtentorial herniation, neuro-worsening, and clinical outcomes, highlighting its clear advantages over subjective pen-light examinations (16–18). In parallel, UPA has emerged as a pragmatic option when optical access is restricted (e.g., eyelid edema, dressings, or ocular injury). Trans-palpebral sonographic approaches have been shown feasible in TBI and periorbital hematoma, providing reliable visualization of the pupil and dynamic traces even through closed eyelids (19, 20).

Beyond static diameters, comparative studies of video/infrared vs. ultrasound pupillometry during nociceptive stimulation indicate that dynamic indices are particularly sensitive to sampling rate, edge-tracking, and timing alignment. This observation is consistent with our findings of small proportional tendencies in velocity metrics and greater between-method variability in latency (21). Notably, automated UPA integrates on-board illumination, edge-tracking, and signal processing, enabling simultaneous extraction of pupil diameter, constriction latency, and velocity metrics (ACV/ADV). As such, it delivers a comprehensive PLR profile comparable to IPA, rather than diameter measurements alone. The somewhat larger variability observed for ACV compared with ADV likely reflects the fact that constriction velocity is driven by the steepest and most rapid portion of the PLR curve, making it more sensitive to frame-to-frame segmentation noise, differences in sampling rate, and small timing misalignments around the peak response. In contrast, dilation tends to be slower and smoother, distributed over a larger number of frames, which may attenuate the impact of single-frame fluctuations and result in narrower limits of agreement for ADV. From a clinical perspective, the observed ACV bias of approximately 0.19 mm/s represents a relative difference of about 13% of the average constriction velocity, which may be acceptable depending on the intended application and decision thresholds. Importantly, no Auto-UPA–related eye discomfort, redness, pain, or other adverse events were observed in this cohort, supporting a favorable safety and tolerability profile for trans-palpebral ultrasound pupillometry in the ICU when performed in accordance with ophthalmic ultrasound safety guidelines and the ALARA (As Low As Reasonably Achievable) principle.

In addition, deploying automated ultrasound systems can substantially reduce clinician workload in high-demand settings. By simplifying acquisition, enforcing standardized workflows, and accelerating on-device data processing, these systems enable clinicians to evaluate more patients efficiently without compromising diagnostic quality. Beyond efficiency, automation also improves safety by allowing reliable use by non-specialists in resource-limited environments where trained personnel are scarce. Moreover, ongoing algorithmic optimization is expected to further enhance image quality and spatial–temporal resolution, thereby increasing diagnostic accuracy. Taken together, these advances establish automated ultrasound as a versatile platform for emergency care, telemedicine, and austere or battlefield conditions, broadening the scope of point-of-care ultrasound as a powerful and flexible tool in neurocritical practice.

## Limitation

Strengths of this study include its prospective, paired ICU design; rigorous, pre-specified control of illumination and testing order; short, fixed inter-test intervals; triplicate averaging; and comprehensive agreement and reliability analyses—approaches recommended in current guidelines and consistent with prior automated pupillometry studies. Limitations primarily relate to the single-center enrollment with a relatively small sample size, and device specificity (EDAN AX8 for ultrasound and a single IPA platform), which may restrict generalizability across different vendors and algorithms. In addition, dynamic-metric comparisons were conducted without hardware-synchronized stimuli, potentially introducing subtle timing discrepancies between Auto-UPA and IPA measurements.

Although we included a predefined reliability subset in which two investigators independently acquired repeated Auto-UPA measurements, the inter- and intra-observer ICCs were derived from this limited two-operator design and may not fully capture variability across a broader range of users or levels of experience. Thus, while the observed ICC values were in the good-to-excellent range, operator-related generalizability beyond our trained ICU team should be interpreted with caution.

Compared with static diameter measurements, dynamic PLR indices such as DELTA and especially LAT are more sensitive to small sources of temporal and segmentation noise. Even minor differences in the identification of constriction onset and peak, frame sampling, or light-trigger alignment between the two devices can translate into larger relative discrepancies in fractional constriction and latency, particularly when absolute diameter changes are modest. These factors likely account for the greater between-method dispersion observed for LAT compared with INIT and END. Notably, between-method dispersion remained small for DELTA in absolute terms, whereas the effect was most pronounced for LAT.

Accordingly, future studies should be multicenter, incorporate standardized stimuli with precise time-base synchronization, and further explore how cross-modality metrics can contribute to predictive frameworks (e.g., integrating automated pupillometry with Doppler-based indices) that are actively under investigation. Future work should also include larger, multi-operator reliability cohorts across different clinical environments to more comprehensively characterize user-dependent variability and external validity.

## Conclusion

Auto-UPA shows strong agreement with IPA for pupil diameters and constriction magnitude, with good-to-excellent inter-observer agreement and robust intra-observer repeatability. Velocity measures were largely concordant, whereas latency proved to be the most method-dependent parameter and should therefore be interpreted with caution across devices. Overall, Auto-UPA offers a rapid, non-invasive, trans-palpebral option for quantitative pupillary assessment when optical access is restricted, and within-device trending remains preferable for dynamic metrics, which appear safe and feasible for bedside use in critically ill patients.

## Data Availability

The original contributions presented in the study are included in the article/supplementary material, further inquiries can be directed to the corresponding authors.
